# Speech Recognition in Noise: Analyzing Phoneme, Syllable, and Word-Based Scoring Methods and Their Interaction with Hearing Loss

**DOI:** 10.3390/diagnostics15131619

**Published:** 2025-06-26

**Authors:** Saransh Jain, Vijaya Kumar Narne, Bharani, Hema Valayutham, Thejaswini Madan, Sunil Kumar Ravi, Chandni Jain

**Affiliations:** 1 Department of Prevention of Communication Disorders, All India Institute of Speech and Hearing, Mysuru 570006, Karnataka, India; thejaswini5342@gmail.com; 2 Department of Audiology, All India Institute of Speech and Hearing, Mysuru 570006, Karnataka, India; bharani2505@gmail.com (B.); chandni.aud@gmail.com (C.J.); 3 Department of Medical Rehabilitation Sciences, College of Applied Medical Sciences, King Khalid University, Abha 61421, Aseer, Saudi Arabia; sravi@kku.edu.sa; 4 Speech-Language Pathology Unit, College of Applied Medical Sciences, King Khalid University, Abha 61421, Aseer, Saudi Arabia; 5 Optisage Technology, Johor Bahru 80730, Johore, Malaysia; hema_valayutham@yahoo.com

**Keywords:** speech perception, hearing loss, auditory processing, scoring methods

## Abstract

**Introduction:** This study aimed to compare different scoring methods, such as phoneme, syllable, and word-based scoring, during word recognition in noise testing and their interaction with hearing loss severity. These scoring methods provided a structured framework for refining clinical audiological diagnosis by revealing underlying auditory processing at multiple linguistic levels. We highlight how scoring differences inform differential diagnosis and guide targeted audiological interventions. **Methods:** Pure tone audiometry and word-in-noise testing were conducted on 100 subjects with a wide range of hearing loss severity. Speech recognition was scored using phoneme, syllable, and word-based methods. All procedures were designed to reflect standard diagnostic protocols in clinical audiology. Discriminant function analysis examined how these scoring methods differentiate the degree of hearing loss. **Results**: Results showed that each method provides unique information about auditory processing. Phoneme-based scoring has pointed out basic auditory discrimination; syllable-based scoring can capture temporal and phonological processing, while word-based scoring reflects real-world listening conditions by incorporating contextual knowledge. These findings emphasize the diagnostic value of each scoring approach in clinical settings, aiding differential diagnosis and treatment planning. **Conclusions**: This study showed the effect of different scoring methods on hearing loss differentiation concerning severity. We recommend the integration of phoneme-based scoring into standard diagnostic batteries to enhance early detection and personalize rehabilitation strategies. Future research must involve studies about integration with other speech perception tests and applicability across different clinical settings.

## 1. Introduction

Speech perception in noise is crucial for effective communication, including social interactions, conveying information, and participation in environments with significant background noise, such as crowded gatherings, public events, or workplaces [1,2]. It serves as a key diagnostic test in audiology. Speech perception in noise depends on several factors, such as the level of background noise [3], type of noise [4], spatial separation of speech and noise [5], reverberation [6], speech characteristics [7], and familiarity [8]. These factors are manipulated in speech perception tests designed to assess an individual’s speech perception ability under noise. Unfortunately, results from these tests are not easily comparable because they are dissimilar in many aspects, such as the type of scoring method they use [9]. This study compared and contrasted various scoring methods, including the phoneme-based, syllable-based, and word-based scoring methods, based on their interaction with the severity of hearing loss. In clinical diagnostics, understanding noise-elicited deficits enhances patient management by tailoring test batteries. Each scoring method elicits unique speech perception features critical for differential diagnosis of auditory processing disorders and hearing loss profiling. A comparison of these methods could provide a thorough insight into the exact mechanisms by which hearing loss alters speech perception at differential linguistic levels.

Phoneme scoring provides insights into bottom-up processing [10], focusing on recognizing individual sounds. From a diagnostic standpoint, phoneme scoring isolates perceptual thresholds at the subphonemic level, offering early markers of cochlear pathology. Studies have shown that phoneme scoring can reduce test result variability by using a larger sample size from the same list of words, offering a clearer view of a listener’s basic auditory discrimination abilities [9]. Furthermore, phoneme scores can be statistically equated for wordlists with iso-phoneme distribution [11]. In contrast, word scoring involves bottom-up and top-down processing [12]. Top-down processing includes higher-level cognitive functions like lexical access and contextual interpretation. Word scoring assesses whole-word recognition, capturing the listener’s ability to use phonetic details and contextual knowledge to understand speech [13]. Clinically, word-based scores mirror real-world communication performance, informing decisions on hearing aid candidacy and rehabilitation focus.

Boothroyd and Nittrouer [14] developed a mathematical model to capture the contextual effect in speech recognition, with the J-factor being one of its parameters. The J-factor measures the relationship between perceptual wholes (e.g., words or sentences) and perceptual parts (e.g., phonemes or words). Diagnostic interpretation of the J-factor can differentiate central versus peripheral auditory deficits. For consonant–vowel–consonant (CVC) syllables, the J-factor is typically approximately 3, indicating that all three phonemes are necessary for recognition. In real-world CVCs, the J-factor is approximately 2.5, suggesting that lexical knowledge helps fill in gaps when phoneme information is incomplete. This lower J-factor value compared to phoneme-based scoring demonstrates how top-down processes, such as lexical access, contribute to more efficient word recognition [15].

Most previous studies have primarily employed monosyllabic words, which do not accurately reflect the linguistic structure of many Indian languages, where bisyllabic words are the minimal meaningful units [16]. In fact, the perceptual scores for bisyllabic words are often higher than those for monosyllabic words, owing to increased contextual cues and linguistic redundancy [17]. This discrepancy raises critical questions about the ecological validity and diagnostic sensitivity of scoring methods based on monosyllables. As a result, there is a persistent need to evaluate different scoring methods, i.e., phoneme, syllable, and word-based scoring, using bisyllabic stimuli, to determine which method most effectively reflects underlying auditory and linguistic processing deficits. This study addresses this gap, particularly in the context of Indian languages, by comparing how these scoring strategies interact with varying degrees of hearing loss.

Comparing scoring across phonemes, syllables, and words promises to refine diagnostic thresholds and improve classification accuracy under varied SNRs, directly informing device fitting and rehabilitation strategies in clinical settings. For example, Sereti et al. [18] explored syllable-based scoring in the Greek Speech-in-Babble test and its effectiveness in children with auditory processing disorder. Schlauch et al. [19] analyzed the impact of list length and phonemic errors on the precision of word recognition scores. Olsen et al. [20] compared phoneme, word, and sentence scoring, discussing the interconnectedness of speech recognition constructs. Valente et al. [21] studied phoneme and word recognition in hearing aid fitting, noting improvements in recognition with programmed-fit versus manufacturer-first-fit settings. Scheidiger et al. [22] assessed hearing aid performance using phoneme tests, assessing the effect of signal processing on speech intelligibility. Schmitt et al. [23] developed a phoneme perception test for high-frequency hearing aid fittings, examining individual differences in phoneme audibility and recognition under various amplification conditions.

Despite these contributions, comprehensive research is still needed to directly compare phoneme, syllable, and word-based scoring methods with hearing loss severity. This study addresses these gaps by evaluating how different scoring approaches impact auditory abilities and speech recognition assessment. By positioning our work within diagnostic frameworks, we aim to refine test protocols and normative reference ranges. We gained valuable insights into these scoring methods by comparing pure tone audiometry thresholds with phoneme, syllable, and word scores in word-in-noise testing. Additionally, discriminant function analysis measured how these scoring methods contribute to differentiating subjects based on their hearing loss severity.

## 2. Materials and Methods

### 2.1. Participants

**Figure 1:**
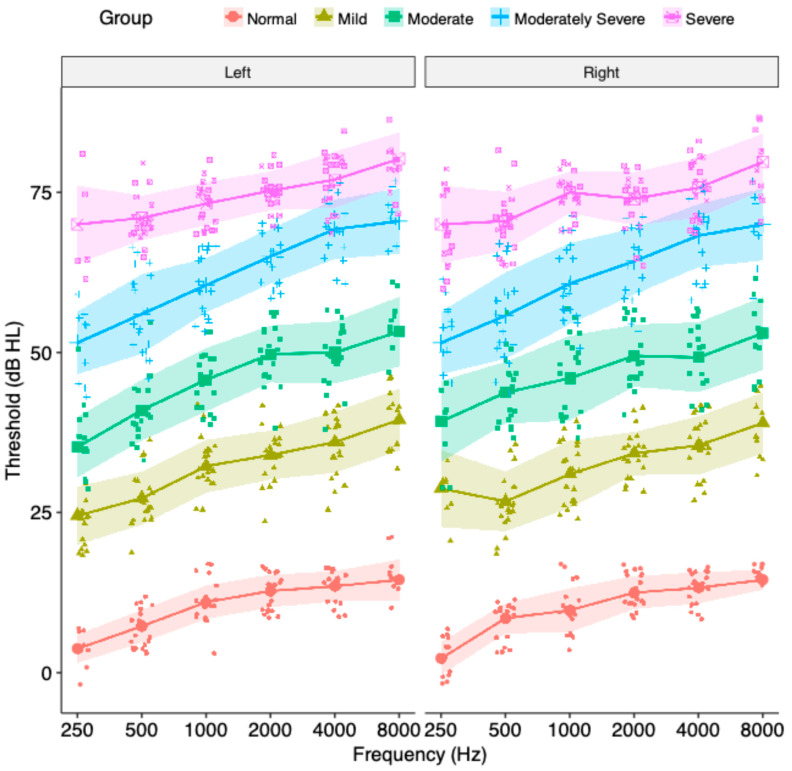
The hearing thresholds of the left and the right ears of subjects in five groups. The solid blocks represent the mean scores. The shaded region along the line joining the mean scores show ± 1 standard deviation. The cluster around the solid block represents the individual data.

One hundred individuals aged 30–40 years volunteered for this study. Participants were divided into five groups (*n* = 20 subjects per group) based on their hearing threshold levels (HTL). All selection criteria adhered to diagnostic standards, ensuring sample representativeness for clinical populations. HTL was determined using pure tone audiometry across frequencies from 250 to 8000 Hz (octave bands) following the modified Hughson-Westlake procedure [24]. According to the ANSI classification system, participants were categorized as having normal hearing or varying degrees of hearing loss [25]. Group 1 subjects had pure tone average (PTA) threshold for 500, 1000, 2000, and 4000 Hz test frequencies ≤ 15 dB HL (normal hearing-reference group), Group 2 subjects had PTA between 26 and 40 dB HL (mild hearing loss), Group 3 subjects had PTA between 41 and 55 dB HL (moderate hearing loss), Group 4 subjects had PTA between 56 and 70 dB HL (moderately severe hearing loss), and Group 5 subjects had PTA between 71 and 90 dB HL (severe hearing loss). Subjects with PTA between 16 and 25 dB (minimal hearing loss) were not included in this study. They were randomly selected from our audiology outpatient department (OPD). Group 1 consisted of individuals accompanying patients, while Groups 2–5 comprised patients visiting the OPD. All selected subjects had binaurally symmetrical hearing with an air-bone gap (ABG) ≤ 10 dB HL at all frequencies. Hearing symmetry was defined as: (a) ≤20 dB difference at any single frequency, (b) ≤15 dB difference at any two adjacent frequencies, and (c) <10 dB difference in PTA at 500, 1000, 2000, and 4000 Hz. All subjects had an uncomfortable loudness level (UCL) > 110 dB HL, measured using recorded speech via the audiometer. Subjects with abnormal loudness growth at suprathreshold levels were excluded. Exclusion criteria were established to ensure the homogeneity of the study sample and minimize confounding variables. The mean hearing thresholds of each group of subjects are shown in Figure 1.

Speech perception scores in quiet aligned with PTA, with speech recognition threshold (SRT) [26] ± 6 dB of PTA. speech identification scores (SIS) were: 90–100% (normal) for Groups 1, 2, and 3; 78–88% (good or slight difficulty) for Group 4; and not less than 66% (fair to moderate difficulty) for Group 5. SRT and SIS were measured following ASHA procedures [27]. Table 1 shows the number of subjects within each group. The table also shows the means and standard deviations (S.D.s), of age, PTA HTLs across 500, 1000, 2000, and 4000 Hz, SRT, and SIS values.

All subjects had ‘A’ type tympanogram [28], indicating no middle ear pathology and normal scores on the Mini-Mental State Examination [29]. Two subjects in Group 2, twelve in Group 3, and all in Groups 4 and 5 were regular hearing aid users. Hearing aids were removed during testing. All procedures were non-invasive and adhered to institutional ethical guidelines. Ethical approval was obtained from the JSSISH ethical committee (no. AP930), and written informed consent was obtained from all participants.

### 2.2. Signal and Noise

Three hundred bisyllabic words (CVCV structure) were selected from a phonemically balanced word list corpus in Kannada for adults [30]. This corpus contains 24 lists, each with 25 words. Twelve lists were randomly chosen for this study. Examples of these CVCV test items include _/di:na/_ (“poor”), _/na:ri/_ (“lady”), _/dura/_ (“far”), and _/bi:di/_ (“country cigarette”), chosen to cover a range of consonant and vowel contexts. The words were recorded by a female native Kannada speaker using the Computerized Speech Lab (CSL) system (Hoya Corporation, Pentax Lifecare, Tokyo, Japan) and digitized through a 16-bit A/D converter at a sampling frequency of 44,100 Hz.

Speech spectrum-shaped noise (SSN) was generated and mixed with each word at specific signal-to-noise ratios (SNRs). The root mean square levels of signal and noise were matched. SSN was added at +5, +2.5, 0, −2.5, −5, and −7.5 dB SNR levels. The noise level varied to achieve different SNRs, with the word level fixed. The noise was processed using custom MATLAB (R2021) code.

Two processed word lists were presented at each SNR level, with no repetitions for the same subject. Therefore, all 12 lists (300 words) were randomly presented (permuted randomization, no repetitions) across six SNR levels. The signal was presented monaurally to the better ear at 80 dB SPL for participants with PTA < 40 dB HL, 90 dB SPL for PTA between 41 and 58 dB HL, and 100 dB SPL for PTA between 59 and 80 dB HL, as per Wilson [31]. This level-specific adjustment was made to ensure audibility across all degrees of hearing loss, while avoiding excessive loudness or discomfort. By adjusting stimulus level based on hearing thresholds, we aimed to maintain a consistent sensation level across participants, thereby reducing the confounding effects of audibility on speech recognition performance. No participant had a PTA exceeding 80 dB HL, thus allowing reliable speech presentation without the need for further amplification. The signal was delivered using a personal computer with Alvin software (ver. 3) [32], routed via a calibrated audiometer (Piano Plus, Inventis Inc., Padova, Italy), and an Etymotic ER-2A insert earphone in a dual-room audiometry setup [33]. Signal processing and SNR manipulations mimic clinical test conditions used in diagnostic audiology labs.

### 2.3. Testing and Scoring

Participants listened to each word and were instructed to repeat it. They were encouraged to guess or repeat the audible sounds if they did not hear or understand the entire word. Responses were recorded using a directional microphone placed 15 cm from the speaker’s mouth, and files were saved in .wav format. Five practice words were provided before testing to familiarize participants with the task, but these practice words were not recorded. Response files were reviewed by three qualified audiologists who rated recordings as correct (score of ‘1’) or incorrect (score of ‘0’) for word, syllable, and phoneme accuracy. Scoring followed the guidelines by Billings et al. [9]. Blinded scoring by three audiologists ensures diagnostic reliability and inter-rater consistency in clinical practice. Word scores were out of 50 per SNR level, syllable scores out of 100 (for bisyllabic words), and phoneme scores out of 200 (four phonemes per word). Inter-judge reliability was very high, with 0% disagreement for word scoring, 0.9% for syllable scoring, and 1.87% for phoneme scoring. The average scores from the three judges were used for further analysis. SNR-50 and SNR-70 were calculated [9], indicating the signal-to-noise ratio at which a listener can correctly recognize 50% and 70% of the words, respectively. These measurements offer quantitative diagnostic thresholds for auditory performance classification.

### 2.4. Data Analysis

Raw scores were converted into percentage correct scores. Statistical thresholds were selected to align with diagnostic decision-making criteria in audiology. SNR-50 and SNR-70 were determined using logistic regression with non-linear interpolation. The effects of scoring systems, SNR measurements, and hearing loss were analyzed using a 3 × 2 factorial repeated measures ANOVA, with post hoc comparisons conducted using Bonferroni corrections. SNR-50 and SNR-70 for phoneme, syllable, and word-based scoring were treated as within-subject variables, while group distribution was the between-subject variable. The correlation between phoneme, syllable, and word-based scoring and PTA was assessed using Pearson’s product-moment correlation. A discriminant function analysis was performed to identify the scoring method that best differentiates between degrees of hearing loss.

## 3. Results

**Figure 2:**
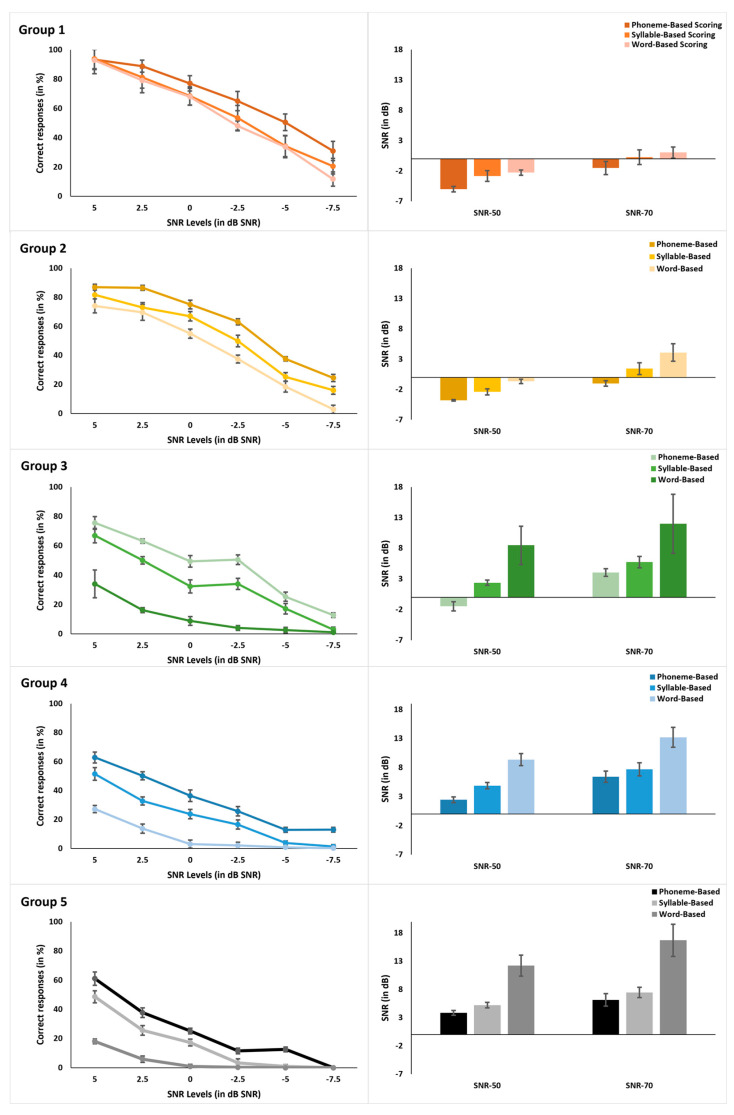
Word accuracy scores (**left panels**) and SNR-50 and SNR-70 values (**right panels**) for phoneme, syllable, and word-based scoring as a function of groups. Error bars show ± 1 standard deviation. Group 1- Normal Hearing; Group 2-Mild; Group 3-Moderate; Group 4- Moderately-Severe; Group 5-Severe Hearing Loss.

Figure 2 (left panels) shows that performance (% correct identification) decreases as SNR decreases across all scoring types, with similar trends observed in all five groups. Correct response percentages were highest for phoneme-based scoring and lowest for word-based scoring. Figure 2 (right panels) presents the SNR-50 and SNR-70 values for phoneme, syllable, and word-based scoring by group. SNR-50 and SNR-70 were lowest for phoneme-based scoring and highest for word-based scoring. Error bars represent ± 1 standard deviation.

A significant effect of type of scoring [F (2, 190) = 839.92, *p* < 0.001, ŋP2 = 0.898] and SNR [F (1, 190) = 1814.91, *p* < 0.001, ŋP2 = 0.950] was noted. The interaction between type of scoring and SNR was also significant [F (2, 190) = 354.52, *p* < 0.001, ŋP2 = 0.789]. Irrespective of the group, the SNR-50 and SNR-70 were lowest for phoneme-based scoring and highest for word-based scoring. A significant effect of hearing loss severity was noted [F (4, 95) = 945.49, p < 0.001, ŋP2 = 0.975]. Group 1 subjects had the lowest SNR-50 and SNR-70 scores, while Group 5 had the highest SNR-50 and SNR-70 scores. The interaction between the type of scoring and group was significant [F (8, 190) = 51.70, *p* < 0.001, ŋP2 = 0.685], indicating that the difference in scores between different scoring types is not uniform across all levels of hearing loss. The interaction between SNR*Type*Group was also significant [F (8, 190) = 40.19, *p* < 0.001, ŋP2 = 0.629], indicating that the effects of scoring type and SNR on performance are interdependent and are moderated by the severity of hearing loss. These statistical outcomes validate the clinical utility of scoring types in diagnostic differentiation.

The SNR-50 and SNR-70 scores were correlated with the subject’s PTA thresholds. Phoneme-based scores maximally correlated with PTA for SNR-50 (r = 0.966, *p* < 0.001) and SNR-70 (r = 0.901, *p* < 0.001), followed by syllable-based SNR-50 (r = 0.927, *p* < 0.001) and SNR-70 (r = 0.892, *p* < 0.001) scores, and least correlated with word-based SNR-50 (r = 0.912, *p* < 0.001) and SNR-70 (r = 0.836, *p* < 0.001) scores. Fisher’s z-transformation was used to compare the correlation coefficients to determine if SNR-50 and SNR-70 for a specific scoring method more strongly correlated with PTA. Table 2 shows the z-transformation and significance values for comparing the correlation coefficients between PTA and each phoneme versus syllable scoring, phoneme versus word scoring, and syllable versus word scoring. Results are shown separately for SNR-50 and SNR-70. The correlation of phoneme-based SNR-50 was significantly higher than syllable and word-based SNR-50 with PTA.

**Figure 3:**
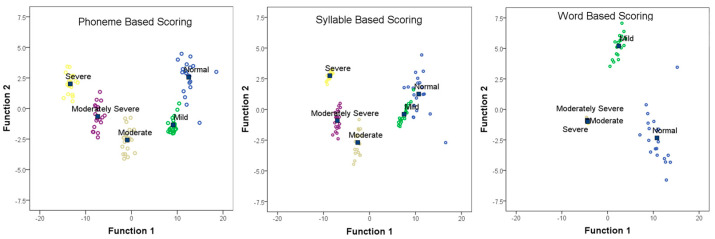
Canonical discriminant function analysis plots for phoneme (left panel), syllable (middle panel), and word-based scoring systems (right panel). Each point represents an individual subject’s response, and the position reflects their scores on two discriminant functions. The scores at each SNR level were the independent variables, and group distribution was the dependent variable. The different colors in each scatterplot represent the five hearing loss severity groups: blue indicates individuals with normal hearing, green represents those with mild hearing loss, yellow denotes moderate hearing loss, purple corresponds to moderately severe hearing loss, and light brown (beige) indicates severe hearing loss. The square boxes indicate the Group Centroid.

Discriminant function analysis assessed the ability of different scoring systems to predict hearing loss severity, with scores at each SNR level as independent variables and group distribution as the dependent variable. Phoneme-based scoring correctly classified 100% of subjects. Four functions were generated; Function 1 explained 94.3% of the variance, while Functions 2, 3, and 4 explained 3.9%, 1.2%, and 0.6%, respectively. Figure 3 (left panel) shows the canonical discriminant function graph, indicating the distinct separation of groups with no overlap, demonstrating phoneme-based scoring’s effectiveness in distinguishing degrees of hearing loss. Syllable-based scoring classified groups with 98% accuracy, with Function 1 explaining 91.9% of the variance. Figure 3 (middle panel) shows some overlap between normal and mild groups, with minor misclassifications, indicating syllable-based scoring’s high accuracy in distinguishing higher degrees of hearing loss but less efficiency between normal and mild hearing loss. One subject with normal hearing was labeled mild, and one with mild loss was labeled normal in the model. Word-based scoring classified groups with 71% accuracy, with Function 1 explaining 83.5% of the variance. Figure 3 (right panel) indicates a high overlap between moderate, moderately severe, and severe hearing loss groups, demonstrating word-based scoring’s efficiency in distinguishing normal and mild hearing loss but poor discrimination for other degrees of hearing loss. Six subjects with moderate hearing loss were identified as moderately severe, and two were labeled severe in the model. Similarly, five subjects with moderately severe hearing loss were identified as moderate, and eight as having severe hearing loss. Two with severe hearing loss were also labeled as moderate, and six as having moderately severe hearing loss in the model.

Further, to see the effect of good and poor SNR in predicting hearing loss severity, we did discriminant function analysis with scores at +5, +2.5-, and 0-dB SNR (good SNR); −2.5, -5-, and −7.5-dB SNR (poor SNR) as the independent variable and group distribution as the dependent variable. Phoneme-based scoring at good SNRs could classify subjects with 88% accuracy and at poor SNRs with 98% accuracy. Syllable-based scoring could classify the subjects with 87% accuracy at good SNRs and 94% accuracy at poor SNRs. Word-based classification could classify the subjects with only 60% accuracy at good SNRs. The statistics could not be computed for poor SNRs as Group 3 to Group 5 subjects could not identify the words at such lower SNRs, resulting in ‘0’ scores for most words. Table 3 shows the predicted group membership at good and poor SNR for phoneme-, syllable, and word-based scoring.

**Figure 4:**
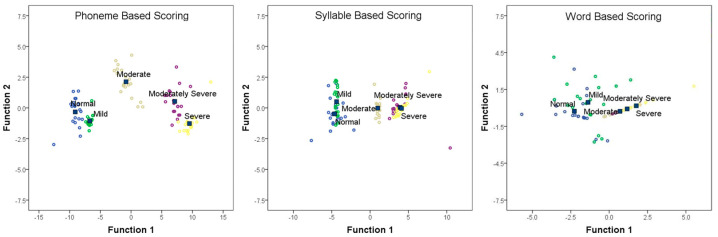
Canonical discriminant function analysis plots for phoneme (left panel), syllable (middle panel), and word-based scoring systems (right panel). The SNR-50 (the signal-to-noise ratio at which 50% of items are correctly identified) and SNR-70 (the signal-to-noise ratio at which 70% of items are correctly identified) scores were the independent variables, and group distribution was the dependent variable. The different colors in each scatterplot represent the five hearing loss severity groups: blue indicates individuals with normal hearing, green represents those with mild hearing loss, yellow denotes moderate hearing loss, purple corresponds to moderately severe hearing loss, and light brown (beige) indicates severe hearing loss. The square boxes indicate the Group Centroid.

Discriminant function analysis further examined SNR-50 and SNR-70 scores as independent variables. Phoneme scoring classified groups with 97% accuracy, with minor misclassifications: two subjects with normal hearing were labeled mild, and one with severe hearing loss was labeled moderately severe. Syllable-based scoring classified groups with 73% accuracy, showing significant overlap in classifying normal and mild, and moderately severe and severe hearing loss. Word-based scoring classified groups with 55% accuracy. Figure 4 shows canonical discriminant functions with SNR-50 and SNR-70 as predictors. Results indicate that phoneme-based scoring, at each SNR level or at SNR-50, is the most efficient method for discriminating hearing loss severity. Clinically, the 100% classification accuracy of phoneme-based scoring supports its adoption as a diagnostic gold standard in hearing loss severity assessment.

## 4. Discussion

The present study evaluates the diagnostic performance of phoneme, syllable, and word-based scoring methods in classifying hearing loss severity, focusing on their classification accuracy and clinical applicability. Our findings demonstrate that phoneme-based scoring achieved 100% correct classification of all subjects, translating to zero misclassifications, whereas syllable-based and word-based methods reached 98% and 71% accuracy, respectively, highlighting phoneme scoring’s superior diagnostic precision. This translates into fewer false negatives in a clinical context, since no subject with hearing loss failed to be correctly identified by phoneme scoring, compared to minor misclassifications observed with syllable and word methods. Phoneme-based scoring minimizes missing data at lower SNRs for hearing-impaired participants, enabling direct estimation of SNR-50 and SNR-70 values without requiring to interpolation. In contrast, word and syllable scoring necessitate non-linear interpolation, which can introduce inaccuracies [34]. By eliminating the need for such approximations, phoneme-based scoring ensures more accurate and reliable estimates. Additionally, phoneme scoring evaluates a higher number of tokens than word and syllable scoring, which in turn reduces variability and increases statistical reliability. This aligns with earlier studies [9,18], demonstrating that the increased dataset size minimizes random errors and enhances sensitivity to subtle differences in speech perception across noise levels or hearing loss severities. Based on group-level misclassification patterns (e.g., one normal misclassified as mild and vice versa with syllable scoring), we suggest defining clinical decision thresholds around phoneme-based SNR-50 values to minimize both false positives and false negatives in diagnostic workflows.

From a diagnostic standpoint, differential reliance on bottom-up (phoneme cues) versus top-down (lexical context) processing may itself serve as a biomarker for central auditory processing disorders. Top-down processes, such as vocabulary knowledge and phonological retrieval, facilitate word identification even when signals are unclear. Clinically, this suggests that word-based scores may overestimate performance in patients who employ strong top-down compensation, whereas phoneme-based scores more directly reflect peripheral encoding fidelity. The J-factor analysis [14] further supports this, showing greater top-down involvement for moderately severe to severe hearing loss at +5 dB SNR compared to normal or moderate loss. The J-factor was estimated using the equation given by Boothroyd and Nittrouer [14]. The J-factor could be estimated only for a +5 dB SNR condition. The average J-factor was approximately 2 for moderately severe and severe hearing loss, whereas the J-factor varied from 1.6 to 1.8 for mild and moderate loss. These observations indicated that there was more top-down involvement for moderately severe to severe conditions at +5 dB SNR and less for normal to moderate hearing loss. For hearing-impaired groups in this study, the J-factor was not available for most of the subjects at SNRs < 5 dB, because they scored below 5% correct on words. Using the J-factor permitted the examination of any potential use of lexical effects during speech perception for SNRs < 5 dB. But at lower SNR, the extent to which the use of lexical effects on word perception is difficult to estimate.

In normal hearing participants, previous studies using behavioral techniques have shown that bottom-up processing is weighted more strongly under conditions such as poor signal-to-noise ratio [35]. Our data confirm that under poor SNRs, phoneme scoring yields a 98% classification rate—versus 94% for syllables and unassessable word scores—highlighting its robustness as a diagnostic measure when audibility is severely compromised. Strauß et al. [36] have shown that, using behavioral and electro-physiological tools, word perception at lower SNRs relies more strongly on bottom-up processing (sub-lexical) due to reduced perceptual evidence in the speech signal. This aligns with our findings, where phoneme-based scoring performed better than syllable- and word-based scoring at lower SNRs, reflecting listeners’ reliance on sublexical features. The differential reliance on sublexical and lexical processing is evident in the significant interactions between SNR, scoring type, and hearing loss severity. At good SNRs, phoneme-based scoring achieved 88% classification accuracy, while syllable- and word-based scoring achieved 87% and 60%, respectively. At poor SNRs, the accuracy of phoneme-based scoring improved to 98%, syllable-based scoring to 94%, while word-based scoring could not be assessed due to the limitations imposed by the noise. Thus, the integration of findings from Strauß et al. [36] provides further support for the importance of sublexical processing in noisy environments. The shift towards sublexical reliance under adverse conditions explains why phoneme-based scoring is particularly effective in low-SNR scenarios.

While our study employed SSN, real-world listening situations involve a variety of maskers, i.e., broadband white noise [37], multi-talker babble [38], and ICRA noise with fluctuating envelopes [39], each imposing different energetic and informational masking demands. White noise affords uniform spectral masking, whereas multi-talker babble (as in HINT or QuickSIN paradigms) adds linguistic interference that taxes top-down processing. ICRA noise captures realistic temporal dips, revealing how listeners exploit momentary “glimpses” of speech. Evaluating phoneme-, syllable-, and word-based scoring across these masker types will test the robustness of phoneme scoring’s diagnostic sensitivity.

Moreover, extending this framework to hearing-device users is critical. Cochlear implant recipients rely heavily on envelope cues and exhibit distinct error patterns on phoneme versus word tasks in noise [40]. Similarly, hearing-aid signal processing strategies (e.g., directional microphones and noise reduction algorithms) differentially affect sub-lexical versus lexical recognition. Integrating phoneme-based scoring with device fitting outcomes could bridge diagnostic audiology and rehabilitative technology, guiding personalized amplification and implant programming.

Beyond pure auditory classification, phoneme-based scoring may also unmask central processing deficits in populations with mild cognitive impairment (MCI). Individuals with MCI often maintain near-normal peripheral thresholds yet struggle with central auditory tasks in noise; a fine-grained phoneme analysis could serve as an early cognitive biomarker.

Furthermore, while our bisyllabic word lists provide sublexical resolution, sentence-level tests such as the Matrix Sentence Test [41] have proven highly effective in evaluating speech intelligibility under realistic noise conditions and are routinely used to assess hearing-aid outcomes [42]. Adapting our scoring framework to Matrix-style stimuli could combine the ecological validity of sentence tests with the diagnostic precision of phoneme-level scoring, offering a powerful tool for both audiological and cognitive assessment.

Despite its high classification accuracy, our study is limited by the use of Kannada bisyllabic stimuli in a controlled lab setting. Future work should validate these discriminant thresholds across different languages, noise types, and real-world clinical environments. Moreover, translating phoneme-based scoring into automated, software-driven tools could expedite diagnostic workflows and reduce examiner bias.

## 5. Conclusions

This study highlights the combined impact of granularity and token quantity in phoneme-based scoring, offering a deeper understanding of its advantages over syllable and word-based methods. By generating a larger dataset and providing finer resolution, phoneme-based scoring improves the accuracy and reliability of speech perception tests, particularly in noisy environments. These findings have practical implications for audiologists and researchers, suggesting that phoneme-based scoring should be prioritized in clinical and research settings. Its superior predictive power for audiometric thresholds and enhanced sensitivity to subtle auditory and cognitive interactions make it a valuable tool for improving the design of speech perception assessments. Integrating phoneme-based scoring within diagnostic protocols can enhance early detection and personalized intervention planning in audiology.

Future researchers should test phoneme-based scoring in broader contexts, such as across tonal and non-tonal language families, in pediatric and geriatric populations, and with ecologically varied noise scenarios (e.g., multi-talker babble and reverberant spaces). Developing user-friendly software platforms that automate phoneme scoring and integrate real-time feedback could streamline clinical workflows and minimize observer variability. Finally, combining phoneme scoring with objective electrophysiological markers (e.g., auditory brainstem responses) and cognitive screening tools may yield a more comprehensive framework for understanding individual differences in speech perception under challenging listening conditions.

## Figures and Tables

**Table 1:** For each group, the overall number of subjects, mean age and standard deviation (SD), and number of males (M) and females (F), the mean and SD values for PTA (pure tone average of HTLs at 500, 1000, 2000, and 4000 Hz), speech recognition thresholds (SRT), and speech identification scores (SIS).

Groups	No. of Subjects	Mean Age (SD)	Gender	Ear	PTA (dB HL)		SRT (dB HL)		SIS (%)	
					Mean	SD	Mean	SD	Mean	SD
**1**	20	33.50 (2.60)	16 M/4 F	*Right*	11.00	1.92	13.25	2.44	97.50	3.44
				*Left*	11.12	1.71	13.50	2.35	97.00	2.99
**2**	20	35.05 (3.77)	15 M/5 F	*Right*	31.87	3.61	34.75	3.79	95.25	3.43
				*Left*	32.37	3.66	34.75	3.79	96.00	3.83
**3**	20	34.80 (3.27)	15 M/5 F	*Right*	47.12	4.44	50.00	4.58	92.75	3.02
				*Left*	46.62	3.62	49.75	4.12	92.25	3.02
**4**	20	35.05 (3.13)	17 M/3 F	*Right*	62.25	4.92	65.25	5.49	82.50	3.44
				*Left*	62.68	4.16	66.25	4.83	82.75	3.02
**5**	20	34.50 (2.89)	16 M/4 F	*Right*	73.81	1.59	76.75	2.44	72.25	3.02
				*Left*	74.12	2.18	78.00	2.51	73.00	2.51

**Table 2:** The z-transformation and significance values for comparing the correlation coefficients between PTA and each of phoneme versus syllable scoring, phoneme versus word scoring, and syllable versus word scoring. Results are shown separately for SNR-50 (the SNR yielding 50% correct recognition) and SNR-70 (the SNR yielding 70% correct recognition).

Comparisons	n	SNR-50		SNR-70	
		z-Values	*p*-Values	z-Values	*p*-Values
phoneme-syllable	100	2.715	<0.001 *	0.322	0.747
phoneme-word	100	3.340	<0.001 *	1.888	0.059
syllable-word	100	0.625	0.532	1.566	0.117

* Values in bold are significant at a 95% confidence interval.

**Table 3:** The predicted group membership at good (+5, +2.5, and 0 dB) and poor (−2.5, −5, and −7.5 dB) SNR for phoneme-, syllable, and word-based scoring. The numbers in each cell represent the number of participants originally belonging to each group (rows) who were classified into each predicted group (columns). Perfect classification would place all values on the diagonal.

Group	Predicted Group Membership									
	Good SNR					Poor SNR				
	Normal	Mild	Moderate	Moderately Severe	Severe	Normal	Mild	Moderate	Moderately Severe	Severe
**Phoneme-Based Scoring**										
**Normal**	10	10	0	0	0	18	2	0	0	0
**Mild**	1	19	0	0	0	0	20	0	0	0
**Moderate**	0	0	20	0	0	0	0	20	0	0
**Moderately Severe**	0	0	0	20	0	0	0	0	20	0
**Severe**	0	0	0	1	19	0	0	0	0	20
**Syllable-Based Scoring**										
**Normal**	10	10	0	0	0	16	4	0	0	0
**Mild**	1	19	0	0	0	2	18	0	0	0
**Moderate**	0	0	20	0	0	0	0	20	0	0
**Moderately Severe**	0	0	0	19	1	0	0	0	20	
**Severe**	0	0	0	1	19	0	0	0	0	20
**Word-Based Scoring**										
**Normal**	10	10	0	0	0	Could not be performed				
**Mild**	1	19	0	0	0					
**Moderate**	0	0	12	6	2					
**Moderately Severe**	0	0	5	7	8					
**Severe**	0	0	2	6	12					

## Data Availability

The data supporting this study’s findings are not publicly available due to copyright permissions from the funding agency, but are available from the author on reasonable request at saranshavi@gmail.com.

## Abbreviations

The following abbreviations are used in this manuscript:

CVC	Consonant–Vowel–Consonant
HTL	Hearing Threshold Levels
PTA	Pure Tone Average
OPD	Out Patient Department
UCL	Uncomfortable Loudness Level
SRT	Speech Recognition Threshold
SIS	Speech Identification Scores
SD	Standard Deviation
CSL	Computerized Speech Lab
SSN	Speech Spectrum Shaped Noise
SNR	Signal-to-Noise Ratio
